# Obese mammary tumour-bearing mice are highly sensitive to doxorubicin-induced hepatotoxicity

**DOI:** 10.1186/s12885-022-10189-z

**Published:** 2022-11-30

**Authors:** Megan Sedeman, Claudia Christowitz, Louis de Jager, Anna-Mart Engelbrecht

**Affiliations:** 1 Department of Physiological Sciences, Stellenbosch University, Stellenbosch Campus, Stellenbosch, South Africa; 2 Department of Global Health, Faculty of Medicine and Health Sciences, African Cancer Institute (ACI), Stellenbosch University, Cape Town, 8000 South Africa; 3 Division of Anatomical Pathology, Stellenbosch University and National Health Laboratory Service (NHLS), Tygerberg Hospital, Cape Town, 8000 South Africa; 4 Anatomical Pathology, PathCare, Cape Town, South Africa

**Keywords:** Obesity, Breast cancer, Doxorubicin, Apoptosis, Non-alcoholic fatty liver disease, Hepatotoxicity

## Abstract

**Background:**

Breast cancer is a major health burden for women, worldwide. Lifestyle-related risk factors, such as obesity and being overweight, have reached epidemic proportions and contributes to the development of breast cancer. Doxorubicin (DXR) is a chemotherapeutic drug commonly used to treat breast cancer, and although effective, may cause toxicity to other organs. The mechanisms and effects of DXR on hepatic tissue, and the contributing role of obesity, in breast cancer patients are poorly understood. The aim of this study was therefore to investigate the effects of DXR on hepatic tissue in an obese tumour-bearing mouse model.

**Methods:**

A diet-induced obesity (DIO) mouse model was established, where seventy-four three-week-old female C57BL6 mice were divided into two main groups, namely the high fat diet (containing 60% kcal fat) and standard diet (containing 10% kcal fat) groups. After eight weeks on their respective diets, the DIO phenotype was established, and the mice were further divided into tumour and non-tumour groups. Mice were subcutaneously inoculated with E0771 triple negative breast cancer cells in the fourth mammary gland and received three doses of 4 mg/kg DXR (cumulative dosage of 12 mg/kg) or vehicle treatments via intraperitoneal injection. The expression levels of markers involved in apoptosis and alanine aminotransferase (ALT) were compared by means of western blotting. To assess the pathology and morphology of hepatic tissue, haematoxylin and eosin staining was performed. The presence of fibrosis and lipid accumulation in hepatic tissues were assessed with Masson’s trichrome and Oil Red O staining, respectively.

**Results:**

Microscopic examination of liver tissues showed significant changes in the high fat diet tumour-bearing mice treated with DXR, consisting of macrovesicular steatosis, hepatocyte ballooning and lobular inflammation, compared to the standard diet tumour-bearing mice treated with DXR and the control group (standard diet mice). These changes are the hallmarks of non-alcoholic fatty liver disease, associated with obesity.

**Conclusion:**

The histopathological findings indicated that DXR caused significant hepatic parenchymal injury in the obese tumour-bearing mice. Hepatotoxicity is aggravated in obesity as an underlying co-morbidity. It has been shown that obesity is associated with poor clinical outcomes in patients receiving neo-adjuvant chemotherapy treatment regimens.

**Supplementary Information:**

The online version contains supplementary material available at 10.1186/s12885-022-10189-z.

## Background

Breast cancer is a common malignancy that frequently occurs in women from both developed and developing countries and is therefore a major health burden worldwide [1]. In 2020, breast cancer was the most frequently diagnosed cancer and the leading cause of cancer-related deaths among females worldwide [2]. Lifestyle-related risk factors, such as obesity and being overweight reached epidemic proportions and is a well-known risk factor that contributes to the development of breast cancer [3]. Dysfunctional adipocytes implicated in obesity is associated with altered secretion of metabolic substrates, adipokines, cytokines, growth factors, and inflammatory molecules which promote cell proliferation, metabolic reprogramming, angiogenesis, invasion and metastasis, and subsequently mediate tumour initiation, progression, and treatment response [4].

Significant progress has been made regarding treatment options for cancer patients, however, these drugs may cause serious multi-organ toxicity, and this remains a major concern. Doxorubicin (DXR) is an anthracycline glycoside antibiotic that possess antitumour activity and is one of the most effective chemotherapeutic agents used to treat breast cancer [5]. Although effective, therapeutic applications of DXR are limited due to its side-effects, such as myelosuppression, chronic cardiotoxicity, and skeletal muscle atrophy [6, 7]. DXR can also be detrimental to other organs, such as the liver, which plays an important physiological role in metabolism, glycogen and triglyceride storage, plasma protein synthesis and detoxification of toxic metabolites [8]. It has been shown that DXR can induce hepatotoxicity, which limits its efficacy in anticancer therapy, resulting in poor patient prognosis and decreased overall survival of cancer patients [9, 10].

DXR can accumulate in the reticuloendothelial system (RES) which is responsible for clearance of particles and substances in circulation and tissues [11]. The RES is a major site of liposome accumulation after systemic administration [11]. The liver is one of the primary organs associated with the RES and exhibits the largest capacity for liposome uptake. Therefore, a substantial amount of DXR can accumulate in liver tissue, making it more prone to DXR-induced toxicity.

DXR-induced oxidative stress is characterized by accumulation of reactive oxygen species (ROS), which decrease antioxidant defence systems, resulting in oxidative damage of deoxyribonucleic acid (DNA), therefore, mediating apoptotic cell death in hepatic tissue [10]. It has been shown that the apoptotic pathway, characterized by the release of cytochrome c from the mitochondria (intrinsic apoptotic pathway) and the Fas ligand (important regulator of the extrinsic apoptotic pathway), were associated with DXR-induced acute toxicity in the liver [10, 12]. Furthermore, histopathological changes have indicated that DXR can cause structural damage to hepatic tissues which include inflammation, congestion, and necrosis [10, 12, 13].

Non-alcoholic fatty liver disease (NAFLD), a co-morbidity associated with obesity is characterized by macrovesicular steatosis, triglyceride accumulation in hepatocytes, increased hepatic oxidative stress and increased sensitivity to drug induced liver injury [14]. A study done by Alghamdi et al*.* (2015) showed that DXR in the presence of palmitate and oleate (lipid loading) caused a significant accumulation of lipids within the human hepatoma cell line (Huh7 cells) and enhanced acute toxicity in lipid-loaded hepatocytes, which is mediated through increased oxidative stress and ROS [15].

The mechanisms of action on hepatotoxicity and the contributing role of obesity in breast cancer patients are not fully elucidated. Therefore, the aim of this study was to investigate the effects of DXR on hepatic tissue in an obese tumour-bearing model. The expression levels of markers involved in the apoptotic pathway was assessed by means of western blotting. Alanine transaminase (ALT) expression was also determined through western blotting. To assess the pathology and morphology of hepatic tissues, haematoxylin and eosin (H&E) staining was performed on formalin fixed paraffin embedded (FFPE) tissue. Furthermore, the presence of fibrosis and steatosis in hepatic tissues were assessed with Masson’s trichrome and Oil Red O staining, respectively.

## Materials and methods

### Animal model

Ethical clearance was obtained from the Stellenbosch University animal research committee (no. SU-ACUM13-00,015 and no. ACU-2020–14,751). The experiments involving the use of laboratory animals were carried out in accordance with the Animal Welfare Act and recommendations of the Institutional Animal Care and Committee of Stellenbosch University. This study is reported in accordance with ARRIVE guidelines (Animal Research: Reporting of In Vivo Experiments) to improve the reporting of research involving animals.

**Fig. 1:**
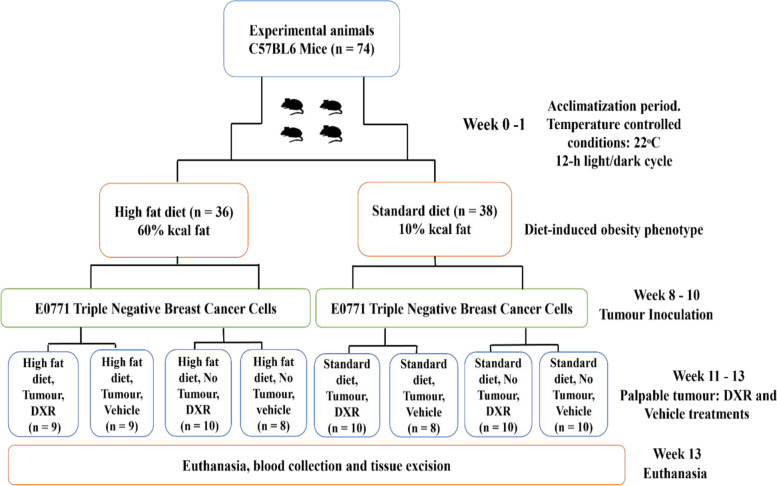
Summary of the in vivo model and respective experimental groups

Three-week-old female C57BL6 mice (*n* = 74) were obtained from the Stellenbosch University Central Research Facility and were housed at the Stellenbosch University Animal Unit in individually ventilated cages (IVC) at temperature-controlled conditions (i.e., 22ºC) and underwent a 12-h light/dark cycle. Mice were allowed an acclimatization period of one week with ad libitum access to mouse pellets and tap water (Fig. 1). The general welfare of the animals was monitored daily. After one week of acclimatization, seventy-four mice were randomly divided into two main groups, namely the high fat diet (HFD, *n* = 36) and standard diet (SD, *n* = 38) groups (Fig. 1). To induce obesity, mice were fed a HFD containing 60% kcal fat (D12492, OpenSource Diets®, Research Diets Inc., New Jersey, USA), whereas a SD containing 10% kcal fat (D12450J, OpenSource Diets®, Research Diets Inc., New Jersey, USA) were used to generate lean control mice (Supplementary data, Table S1) [16]. According to literature, C57BL/6 mice are sensitive to diet-induced obesity (DIO) [17]. Body weight was monitored weekly over the study period and mice were on their respective diets up until the end point of the study.

After eight weeks on their respective diets, the DIO phenotype was established and the two groups were further randomly divided into two groups each, namely the tumour (T) and non-tumour (NT) groups. Mice were subcutaneously inoculated with E0771 triple negative breast cancer cells suspended in Hanks Balanced Salt Solution (HBSS) (Sigma Chemical Co., St Louis, MO, USA) in the fourth mammary pad, using a 23-guage needle syringe. The tumour groups were inoculated with E0771 triple negative breast cancer cells (HFD + T, *n* = 18; SD + T, *n* = 18) and the non-tumour groups were not inoculated with cancer cells (HFD + NT, *n* = 18; SD + NT, *n* = 20) (Fig. 1).

Once the tumours became palpable (200 – 300 mm^2^), DXR treatment was initiated. The mice were randomly divided into vehicle control (V, isovolumetric intra-peritoneal injection of HBSS) and DXR treatment (D5794, LKTR laboratories, Minnesota, USA) groups. Mice were restrained and three successive dosages of 4 mg/kg DXR were administered every three days (cumulative dosage of 12 mg/kg) via intraperitoneal injection. In humans, the dosage of 12 mg/kg DXR is equivalent to 36 mg/m^2^ and falls within the relevant dosage range of DXR treatment (15—90 mg/m^2^) administered to cancer patients in the clinical setting [18]. The eight experimental groups were assigned as follow: High fat diet + Tumour + Doxorubicin (HFD + T + DXR, *n* = 9); High fat diet + Non-Tumour + Doxorubicin (HFD + NT + DXR, *n* = 10); Standard diet + Tumour + Doxorubicin (SD + T + DXR, *n* = 10); Standard diet + Non-Tumour + Doxorubicin (SD + NT + DXR, *n* = 10), High fat diet + Tumour + Vehicle (HFD + T + V, *n* = 9); High fat diet + Non-Tumour + Vehicle (HFD + NT + V, *n* = 8); Standard diet + Tumour + Vehicle (SD + T + V, *n* = 8); Standard diet + Non-Tumour + Vehicle (SD + NT + V, *n* = 10) (Fig. 1).

The mice were weighed every second day and the last body weight was recorded on the day of euthanasia. Tumour growth was measured using a Harpenden caliper (mm) and individual tumour volumes were calculated according to the following equation [19]:

$$Tumour\;volume\;(mm^3)=\;\frac1{2\;\left(length\;x\;width^2\right)}$$

Fasting blood glucose, triglyceride and lactate levels were taken using the tail prick method where 5 μl of blood was collected on test strips using the Accu-Chek ® Performa Nano (Roche Diagnostics, Mannheim, Germany) and Accutrend Plus® (Roche Diagnostics, Mannheim, Germany) respectively. The mice were euthanized 3 days after the last DXR treatments were administered. The mice were anesthetized with 3% isoflurane (Isofor, Safeline, Pharmaceuticals, Florida, South Africa) and were euthanized by cervical dislocation. Hepatic tissues were excised, where half of the tissues were snap frozen in liquid nitrogen and stored at—80 °C for western blotting and Oil red O staining (*n* = 4). The other half of the tissues were preserved in 10% formalin for histological analysis (*n* = 4–5).

### Blood analysis

Blood plasma samples were used to quantify TNF-a, IL-6, IL-10, leptin (PPX-04-MXCE327, Thermo Fisher Scientific, United States) using a custom ProcartaPlex panel and matched mouse Luminex kit. Insulin was quantified using a Milliplex mouse adipokine magnetic bead panel MAP kit (MADKMAG-71 K, Burlington, Massachusetts, United States). All analyses were performed according to the manufacturers’ protocols and specifications. Analytes were measured simultaneously using a MAGPIX system plate reader (APX1042, Bio-Rad, California, United States) and data (expressed in pg/ml) was processed on Bioplex Software 6.1 (Bio-Rad, California, United States).

### Western blot

Hepatic tissue samples were placed on ice and allowed to thaw. Samples were suspended in 300 μl cold modified radio-immunoprecipitation assay buffer (RIPA) containing protease and phosphatase inhibitors. Surgical scissors cleaned with 100% ethanol was used to cut tissues into smaller pieces while on ice. Samples were homogenised (KineMatica PolytronTM PT2100, Fisher Scientific) while on ice. Samples were centrifuged (14, 000 RCF (g), 20 min, 4 °C) to yield distinct layers and the supernatant layer was removed and transferred into sterile Eppendorf tubes. Samples were then centrifuged again at 14, 000 RCF (g), 20 min, 4 °C. The process of removing the supernatant was repeated followed by protein determination using a Bradford assay. Protein samples were prepared with Laemmli’s sample buffer and were loaded onto 4–20% Criterion™ TGX Stain-Free™ Precast Gels (mini-PROTEAN® TGX™ Gels, Bio-Rad), following protein separation at 100 V for 10 min and 120 V for 60 min in Tris/Glycine/SDS running buffer (BioRad, CA, USA). Proteins were transferred onto Polyvinylidene difluoride (PVDF) membranes (Trans-Blot Turbo RTA Midi PVDF transfer kit, BioRad, CA, USA) with the Trans-Blot Turbo Transfer System (BioRad, CA, USA) using mixed molecular weight. The membranes were blocked in 5% milk prepared in tris-buffered saline with tween 20 (TBS-T) for 2 h at room temperature (RT) and then incubated in primary antibody, at 4 °C overnight. On the following day, the membranes were incubated with secondary antibody for 1 h at RT. Primary and secondary antibody details are listed in the additional file (Supplementary data, Table S2). After incubation, the membranes were developed on the ChemiDoc™ MP System. Specific bands were visualized and detected using enhanced chemiluminescence (ECL) substrate detection (BioRad, CA, USA). Quantification of protein samples were normalized to total protein and expressed as a percentage of the control.

### Histology

In all animals, a small portion of the right lateral lobe of the liver tissue was fixed in 10% neutral formalin buffered solution prior tissue processing (*n* = 4–5). Hepatic tissues were processed using an automated tissue processor (HistoCore PEARL, Leica Biosystems) on a 12-h cycle followed by infiltration with paraffin embedded wax (Leica EG 1150 H). Tissues were sectioned into 5 μm sections using a microtome (Leica RM 2125 RT) and tissue sections were placed onto positively charged histobond microscope slides. Two histochemical stains were carried out on the sectioned tissue; H&E stain for morphometric and pathological evaluation and Masson’s trichrome stain to evaluate the presence of fibrosis within hepatic tissue. The right median lobe of hepatic tissue was snap frozen in liquid nitrogen and sectioned into 7 μm sections using a cryostat (Leica CM 3050 S Research Cryostat, Leica Biosystems) to evaluate the presence of lipid accumulation using Oil red O staining (*n* = 4–5). Sections were placed onto positively charged histobond microscope slides and left to defrost. Sections were stained in Oil red O in dextrin staining solution (Sigma-Aldrich, 01,391, SA) for 25 min. Coverslips were mounted onto the slides using aqueous mounting media (Sigma-Aldrich, G 0918) prior to imaging under a microscope (Nikon ECLIPSE E400). The relative number of red pixels (lipid droplets) were quantified using Image J software v1.52a.

### Histopathology

Three individuals, blinded to the treatment allocations, scored five images per tissue sample in each treatment group using the non-alcoholic fatty liver disease (NAFLD) activity score (NAS). This is an accredited and validated scoring system frequently used to evaluate hepatic steatosis, inflammation, and hepatocyte-specific pathology [20]. Each scorer was provided with a grading sheet to familiarize themselves with the scoring system one day prior to analysis. The scores were tabulated and used for inter- and intra-observer analysis. H&E-stained tissues were used to evaluate structural changes that occurs within hepatocytes and Masson’s trichrome-stained tissues were used to assist with the evaluation of liver fibrosis. Steatosis was scored according to its percentage per microscopic field, location (zone, 1, 2 or 3), and the presence of micro- and/or macrovesicular steatosis. With regards to inflammation, the location, lobular and/or portal, were evaluated per 100X microscopic field. Following H&E staining, microvesicular steatosis were indicated by black arrows and macrovesicular steatosis were indicated by red arrows (Fig. 5). Ten images per sample were taken and analysis was performed from right to left across the liver tissue sections. The H&E-stained and Masson’s trichrome-stained liver tissues were evaluated as follow: To assess the extent of steatosis, the presence of hydropic changes, micro- and macrovesicular steatosis were evaluated. To determine whether inflammation occurred, the presence of portal mononuclear cell infiltration, haematopoiesis, and Kupffer cell proliferation were evaluated. Characteristics of hepatocytes and the portal system, such as hepatocyte swelling, sinusoidal and central vein dilation, were also evaluated.

### Statistical analysis

The western blot experiments were conducted with biological repeats of *n* = 4 and technical repeats of *n* = 1. Bio-Rad Image Lab™ software v6.0.1 was used for normalization of the protein specific intensities against total protein intensities. For the Oil Red O staining, ten images per sample were quantified in Image J software v1.52a and the relative number of red pixels were analysed among the different treatment groups. Statistical analysis was performed using GraphPad Prism v7.0. To determine whether the data was normally distributed, a normality test was performed using the Shapiro-Wilks test. To describe the differences between two groups, a Mann Whitney t-test was used. A three-way ANOVA (analysis of variance) followed by Fishers LSD post hoc test was used to describe the differences between three/or more groups and to determine the relationship between the three variables present in this study, namely, diet, disease, and treatment. The results were reported as mean ± standard error of the mean (SEM) and *p* < 0.05 was considered statistically significant. Histological experiments were conducted with biological repeats of *n* = 4–5 and technical repeats of *n* = 2.

## Results

### Establishing a diet-induced obesity phenotype

**Fig. 2:**
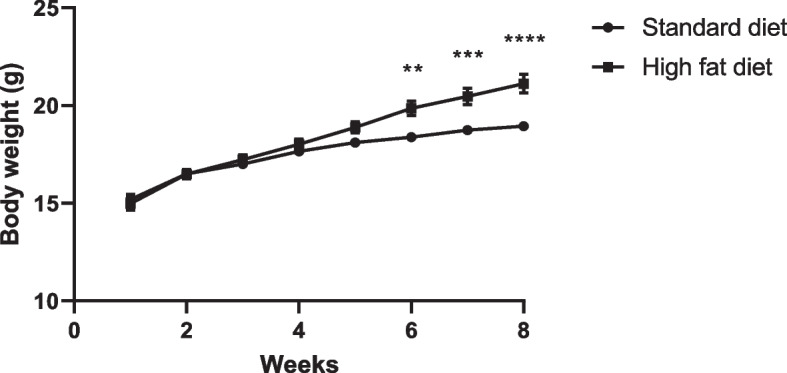
Body weight of female C57BL6 mice from week one to week eight on their respective diets. A Mann Whitney t-test was performed to compare the differences in body weight (grams) between the SD and HFD mice after eight weeks. Values were represented as mean ± SEM and *p* < 0.05 was considered statistically significant (SD, *n* = 38; HFD, *n* = 36). **—*p* < 0.01, ***—*p* < 0.001, ****—*p* < 0.0001. SD: standard diet; HFD: high fat diet

To establish whether obesity was induced, differences in body weights were determined between mice fed a SD and HFD for eight weeks, prior to tumour inoculation and DXR treatment (Fig. 2). After eight weeks on their respective diets, mice fed an HFD showed statistically significant higher body weights at week 6 (*p* < 0.01), week 7 (*p* < 0.001) and week 8 (*p* < 0.0001) compared to mice fed a SD **(**Fig. 2). We therefore conclude that the DIO phenotype was established after 8 weeks on their respective diets and continued throughout the study.

**Fig. 3:**
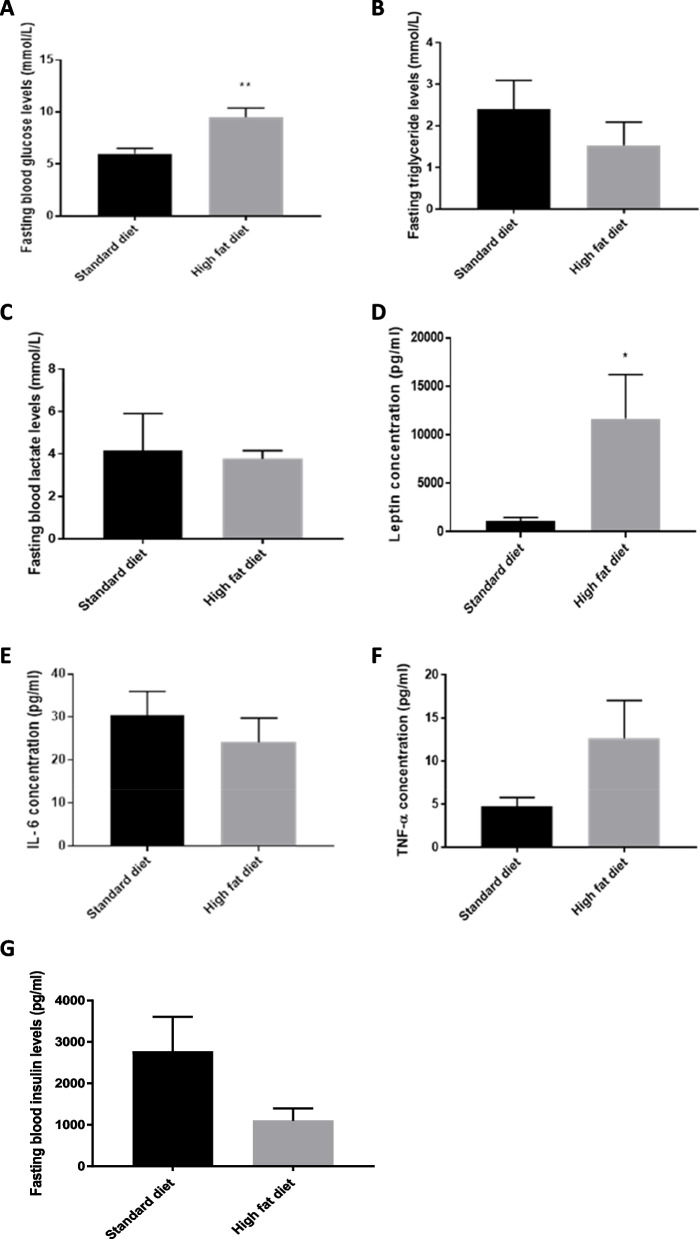
Fasting blood parameters of mice fed a standard or high fat diet for ± 12 weeks. Mice were fasting for 12 h prior to euthanasia to determine (**A**) glucose, (**B**) triglycerides, (**C**) lactate, (**D**) leptin level (**E**) interleukin-6, (**F**) TNF-α and (**G**) insulin levels. Results are represented as mean ± SEM (*n* = 5–6). *—significantly different compared to the standard diet group (*p* < 0.05). **—significantly different compared to the standard diet group (*p* < 0.01)

To further confirm the DIO phenotype, fasted blood parameters such as fasted glucose, triglyceride, lactate, and insulin levels were determined. Adipokines, such as leptin, IL-6 and TNF-α were also assessed. No statistically significant differences were observed for triglycerides (Fig. 3B), lactate (Fig. 3C), insulin (Fig. 3G), IL-6 (Fig. 3E), and TNF-α (Fig. 3F) levels between the different treatment groups. Fasting blood glucose (*p* = 0.043) (Fig. 3A) and leptin levels (*p* = 0.0267) (Fig. 3D) were significantly up regulated in the HFD group compared to the SD group.

### Hepatic tissue weight and hepatic hypertrophy

**Fig. 4:**
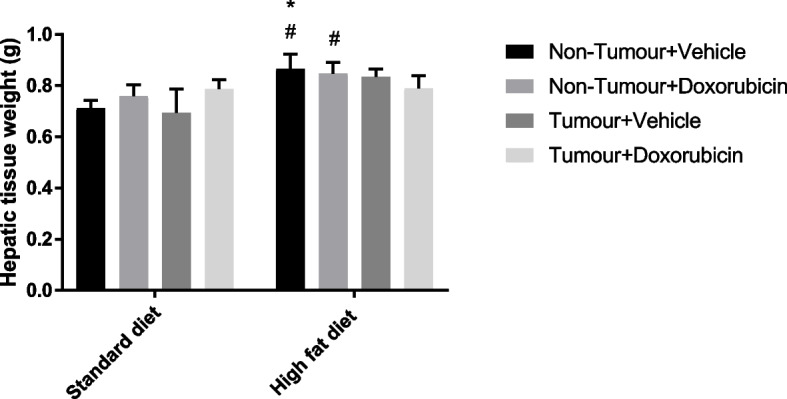
Hepatic tissue weight (grams) between the different groups. Three-way ANOVA with Fishers LSD post hoc correction test was applied and *p* < 0.05 was considered statistically significant. Results are presented as mean ± SEM (*n* = 8–10 per group). *—significantly different compared to the SD + NT + V group (*p* < 0.05), #—significantly different compared to the SD + T + V group (*p* < 0.05). SD: Standard diet; HFD: High fat diet; T: Tumour; NT: Non-Tumour; DXR: Doxorubicin; V: Vehicle

Mice in the HFD + NT + V group showed a significantly higher hepatic tissue weight compared to the SD + NT + V mice (*p* = 0.0406) and SD + T + V mice (*p* = 0.0218) (Fig. 4). Mice in the HFD + NT + DXR group showed a significantly higher hepatic tissue weight compared to mice in the SD + T + V group (*p* = 0.0400) (Fig. 4).

### Hepatotoxicity: Apoptotic cell death and ALT

For the purpose of this study, we focussed on the three objectives namely, 1) the effects of obesity on hepatotoxicity, 2) the effects of DXR treatment on hepatotoxicity, and 3) the effects of obesity on the outcomes of DXR-induced hepatotoxicity. All western blot results are included in the supplementary data set.

### Apoptotic cell death

To determine whether apoptosis was induced in the hepatic tissue samples, western blot experiments were performed to compare the protein expression levels of different apoptotic markers between the different treatment groups. For the intrinsic apoptotic pathway, no significant differences were observed in caspase-9 protein expression between the different treatment groups (supplementary data, Fig. S1). We also assessed the effector caspase, where no significant differences were observed in caspase-3 protein expression between the different treatment groups (supplementary data, Fig. S4). Furthermore, no significant differences were observed in cleaved PARP protein expression between the different treatment groups (supplementary data, Fig. S5). There were also no significant differences observed in ALT expression between the different treatment groups (supplementary data, Fig. S6).

### Histopathology

#### Histopathology of hepatic tissue (H&E staining)

**Fig. 5:**
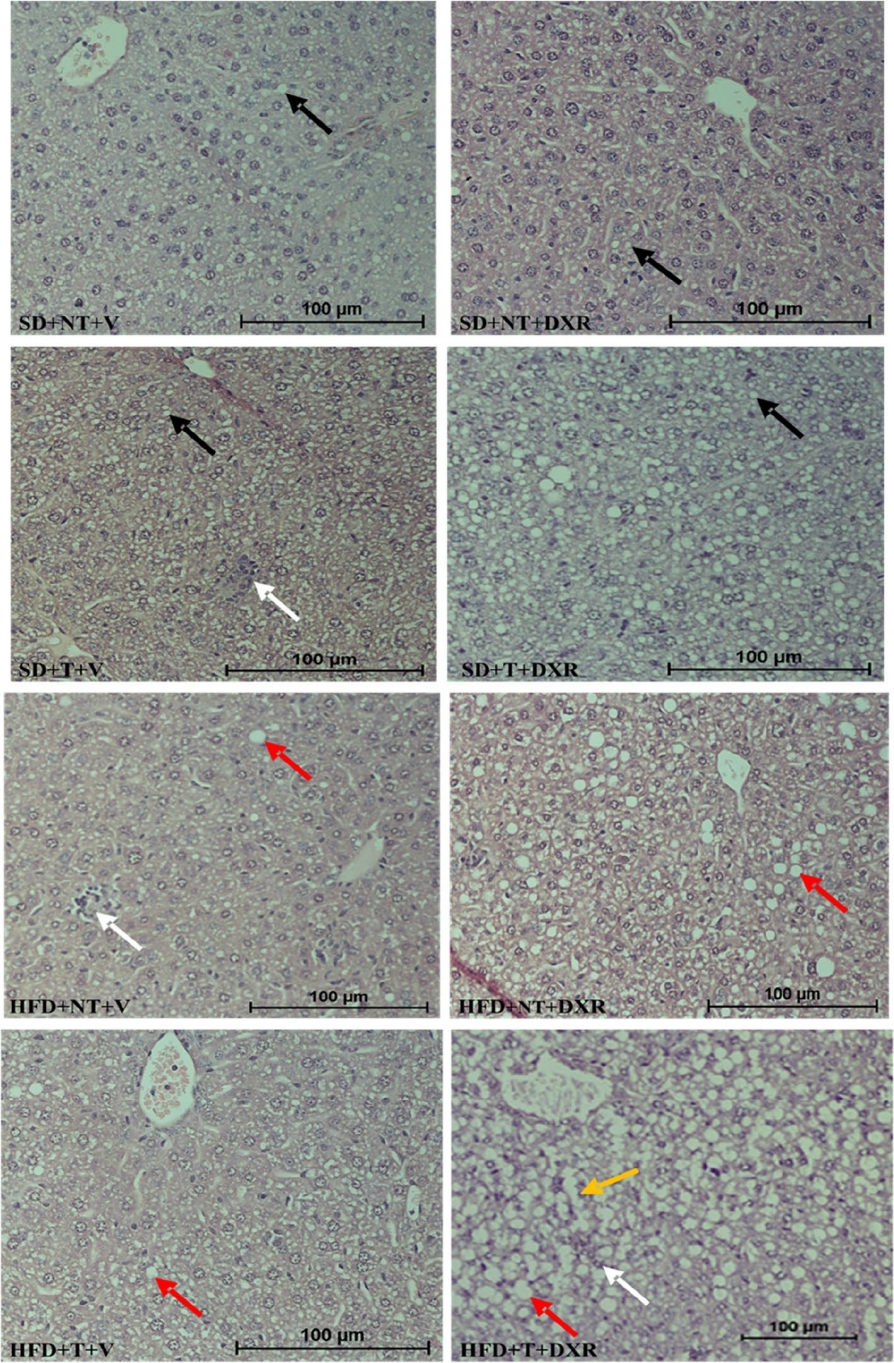
Examples of pathological lesions observed in H&E-stained hepatic tissue in mice fed a HFD evaluated by the non-alcoholic fatty liver disease activity scoring system (NAS) (*n* = 4–5). Microvesicular steatosis (indicated by black arrows) and macrovesicular fatty infiltration in hepatocyte (steatosis) (indicated by red arrows), mild inflammation (inflammatory foci) (indicated by white arrows) and sinusoidal dilation (indicated by yellow arrow) within hepatocytes between the different groups. Images were taken at 20 × magnification, scale bar = 100 μm

Using the accredited NAFLD activity score (Table 1), macrovesicular steatosis was observed in the HFD + NT + DXR group with > 33%-66% steatosis present (grade 2). The HFD + T + DXR group showed extensive hepatocyte ballooning and sinusoidal dilation with > 66% steatosis present (grade 3) compared to the SD + NT + V (control group) where areas of steatosis alternated with areas of normal morphology (Table 1). Macrovesicular steatosis were therefore observed in the HFD + T + DXR group (indicated by red arrow) accompanied with mild inflammation (indicated by white arrow) and sinusoidal dilation (indicated by yellow arrow) and congestion (Fig. 5). Vacuoles of lipids are accumulating in the hepatocytes in the HFD + T + DXR group and are displacing the nucleus to the cell’s periphery (indicated by red arrow) (Fig. 5). Inflammatory foci (mild inflammation) were observed in the SD + T + V, HFD + NT + V and HFD + T + DXR groups (indicated by white arrow) with < 2 foci per 200 × field (grade 1) (Fig. 5, Table 1).

**Table 1:** Liver pathology evaluated by the nonalcoholic fatty liver disease activity score [20, 21]

Components of nonalcoholic fatty liver disease activity score		
Item	Definition	Score
Steatosis	< 5%	0
	5%—33%	1
	> 33%—66%	2
	> 66%	3
Lobular inflammation	No foci	0
	< 2 foci per 200 × field	1
	2–4 foci per 200 × field	2
	> 4 foci per 200 × field	3
Ballooning	None	0
	Few balloon cells	1
	Many cells/ prominent ballooning	2

Macrovesicular steatosis was minor in the HFD + NT + V group (indicated by red arrow) and accompanied with mild inflammation (indicated by white arrow) (Fig. 5). Macrovesicular steatosis was moderate in the HFD + NT + DXR group (indicating in red arrow), which indicates that DXR aggravates the toxicity in hepatic tissue (Fig. 5). Macrovesicular steatosis was minor in the HFD + T + V group (indicated by red arrow) (Fig. 5). Microvesicular steatosis with < 5% steatosis within hepatocytes occurred in the SD + NT + V control group (grade 0), accompanied with no inflammatory foci (grade 0) and none ballooning present (grade 0) (indicated by black arrow) (Fig. 5, Table 1). Microvesicular steatosis with > 33%-66% steatosis within hepatocytes were present in the SD + T + DXR group (grade 2), accompanied with no inflammatory foci (grade 0) and few balloon cells (grade 1) (indicated by black arrow) (Fig. 5, Table 1). Macrovesicular steatosis with 5%-33% steatosis within hepatocytes (grade 1), accompanied with inflammatory foci (grade 1) (indicated by white arrow) and non-ballooning were observed in the HFD + NT + V group, where areas of steatosis > 33%-66% were observed in the HFD + NT + DXR group with no inflammatory foci present (grade 0) (Fig. 5, Table1). Macrovesicular steatosis 5%-33% were observed within hepatocytes in the HFD + T + V group (grade 1) (indicated by red arrow) (Fig. 5, Table 1). To summarise; our results demonstrated that macrovesicular steatosis was observed in mice fed a HFD and microvesicular steatosis was observed in mice fed a SD.

#### Oil red O staining

**Fig. 6:**
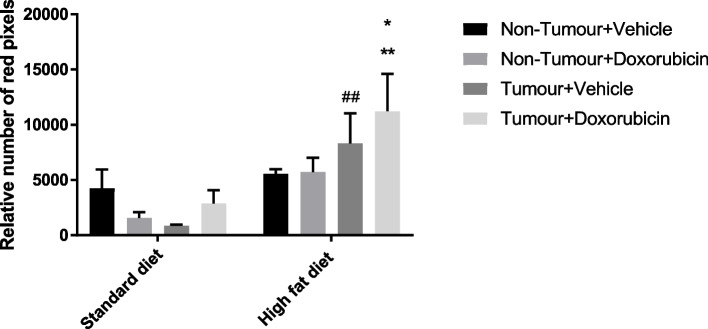
Relative number of red pixels indicating the amount of lipid droplets within hepatocytes (*n* = 4–5). Results are presented as mean ± SEM. Three-way ANOVA with Fishers LSD post hoc correction test was applied and *p* < 0.05 was considered statistically significant. *—Significantly different compared to the Standard diet + Tumour + Doxorubicin (SD + T + DXR) group (*p* < 0.05), **—Significantly different compared to the SD + T + DXR group *(p* < 0.01), ##—Significantly different compared to the Standard diet + Tumour + Vehicle (SD + T + V) group (*p* < 0.01). SD: Standard diet; HFD: High fat diet; T: Tumour; NT: Non-Tumour; DXR: Doxorubicin; V: Vehicle

**Fig. 7:**
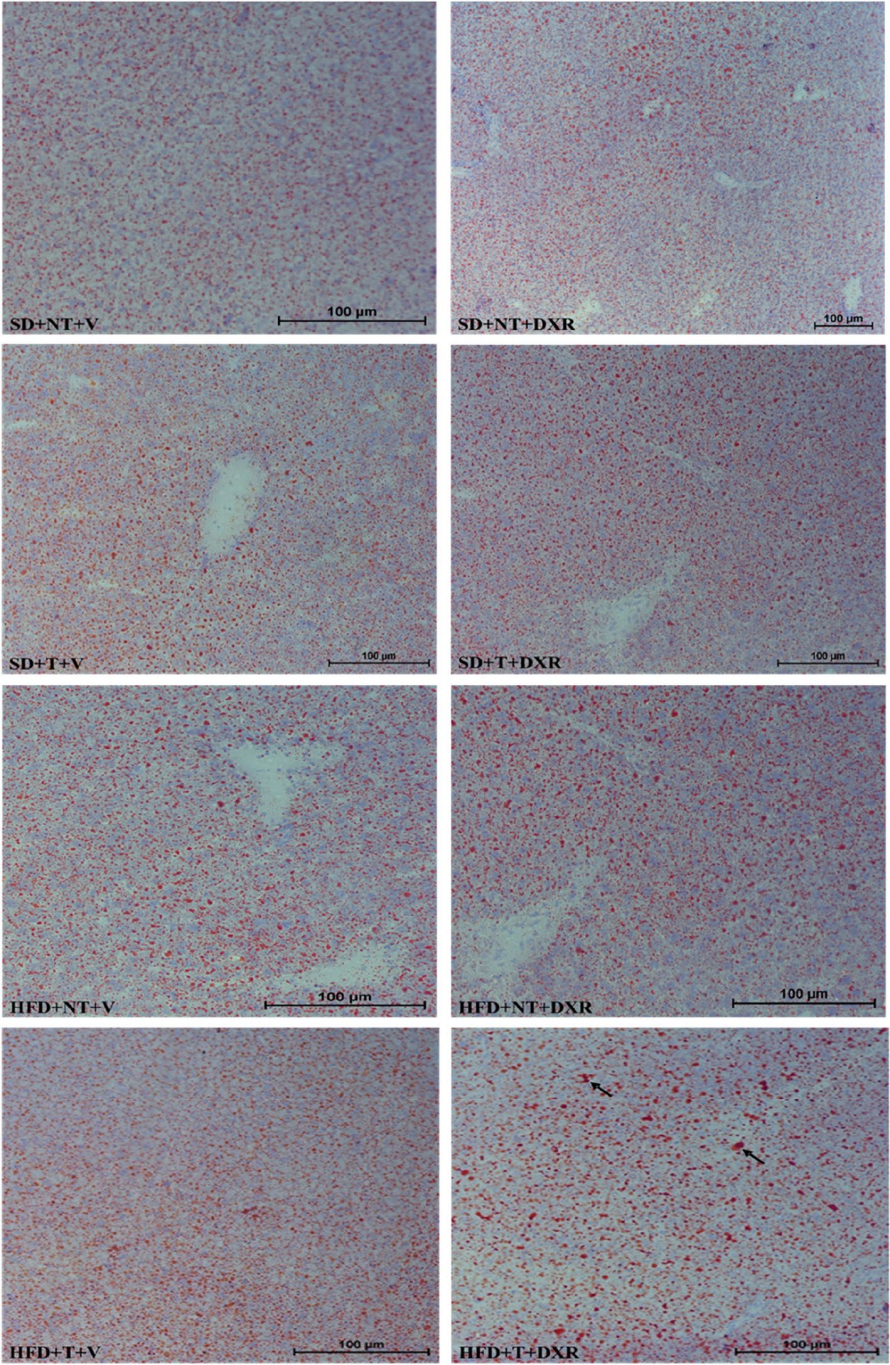
Representative images of lipid accumulation in hepatic tissue detected by the Oil Red O stain (*n* = 4–5). Lipid droplets (indicated by black arrows). Images were taken at 20 × and 10 × magnification, scale bar = 100 μm

To evaluate the presence of lipid accumulation within hepatocytes, Oil Red O staining was performed (Fig. 7). To detect the amount of lipid droplets among the different treatment groups, the relative number of red pixels were analysed using Image J (Fig. 6). This method was used by Masone et al., 2017 [22] & Gojanovich et al., 2016 [23] to accurately quantify and analyse size distribution of cellular lipid droplets. The HFD + T + V group showed a highly statistically significant increase in the relative number of red pixels (lipid droplets) compared to the SD + T + V group (*p* = 0.0057). The HFD + T + DXR group indicates a highly statistically significant increase in the relative number of red pixels (lipid droplets) compared to the SD + T + DXR (*p* = 0.0023) and the HFD + NT + V control group (*p* = 0.0318). Hepatocytes stain amphophilic and lipid droplets are stained red (indicated by black arrows) (Fig. 7). High levels of lipid accumulation were observed within hepatocytes in the HFD groups. Furthermore, lipid accumulation was significantly increased in the HFD group treated with DXR and the tumour present, which indicates that lipid accumulation was more severe in the HFD + T + DXR group compared to the SD + T + DXR group (Figs. 6 and 7).

#### Masson’s trichrome stain

**Fig. 8:**
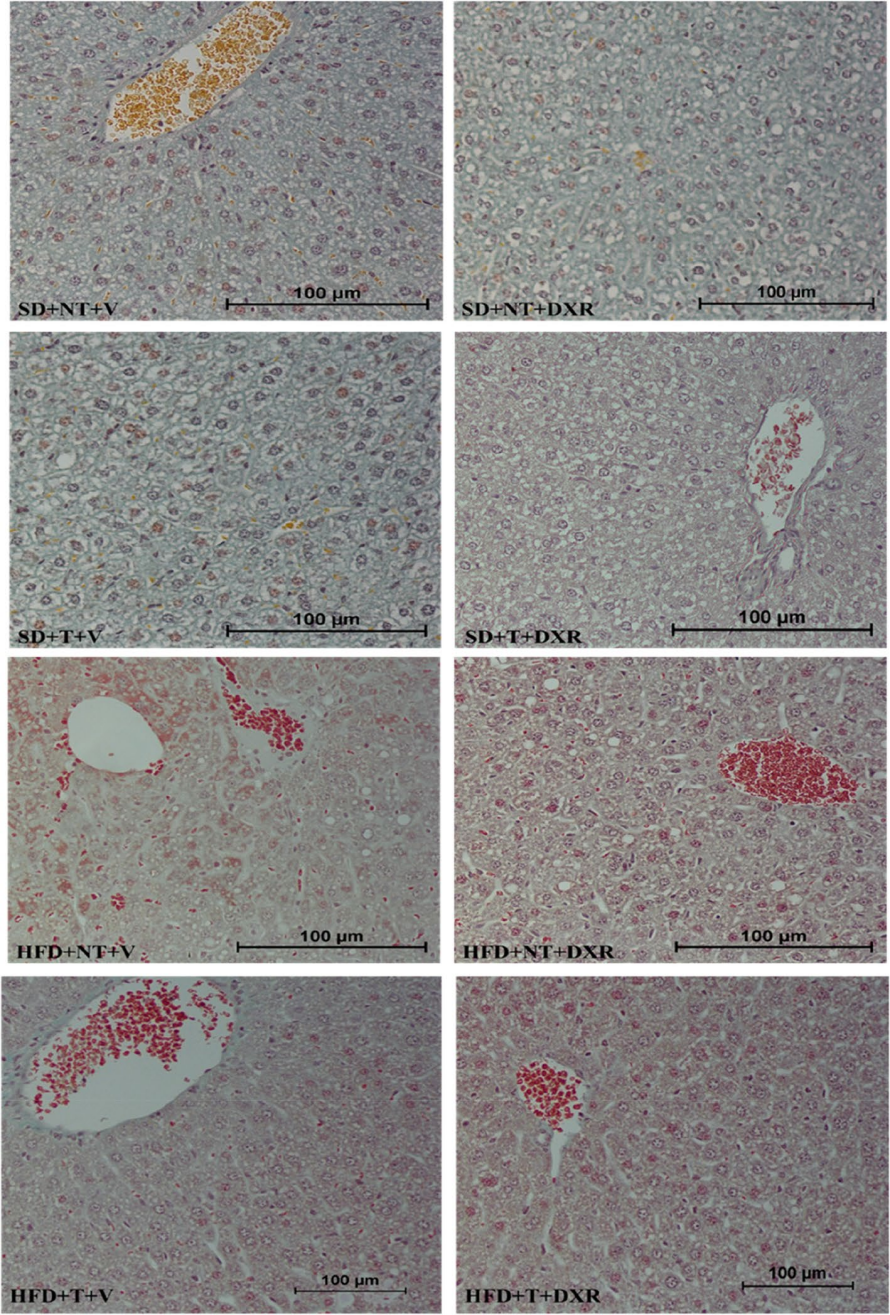
Representative images of liver tissue stained by the Masson’s trichrome stain for pathological evaluation of fibrosis. (*n* = 4–5). Images were taken at 20 × and 40 × magnification, scale bar = 100 μm

To evaluate liver damage, collagen fibres are stained blue using Masson’s trichrome stain to determine the presence of fibrosis. No significant fibrosis was observed within the treatment groups, and therefore, no trends or statistical significance could be determined (Fig. 8). Images were qualitatively represented.

## Discussion

### Establishment of an obese model following a high fat diet

In animal models, a HFD is associated with an increase in body mass (BM) [24–27] and liver mass, which was observed in the HFD groups within this study. To establish whether obesity was induced, differences in body weight was evaluated for mice fed a SD or HFD for eight weeks prior to tumour inoculation and DXR treatment (Fig. 2). The body weights of mice in the HFD group were significantly higher compared to the SD group (Fig. 2), which confirmed that the obese phenotype was successfully established in our in vivo model. Our results correlate with similar findings of a study done by Lee et al*.* (2019) [28] and Santander et al. (2015) [29] who indicated that a HFD increased body weight, as a result of increased fat mass in C57BL6 mice by evaluating body composition. It has been shown that the C57BL6 mice strain is highly susceptible to the development of DIO and therefore correlates with the findings of our study [17].

To further establish the DIO phenotype, fasted blood parameters such as fasted blood glucose (Fig. 3A), triglycerides (Fig. 3B), lactate (Fig. 3C), and insulin levels (Fig. 3G) were determined. Adipokines, such as leptin (Fig. 3D), IL-6 (Fig. 3E) and TNF-α (Fig. 3F) were also assessed. While no statistically significant differences were observed for triglycerides, lactate, IL-6 and TNF-α levels, fasting blood glucose (*p* = 0.043) and leptin (*p* = 0.0267) levels were significantly up-regulated in the HFD group compared to the SD group. No significant differences were observed in the insulin levels between the different groups (Fig. 3G). Obesity is associated with insulin resistance which can promote the development of type 2 diabetes mellitus (T2DM). Therefore, the elevated fasting blood glucose levels in the HFD group is possibly indicative of insulin resistance associated with T2DM, clinically evidenced as a hyperglycemic state. The increased leptin levels observed in the HFD group, further confirmed the state of obesity. Leptin, secreted by adipocytes, increases with higher degrees of adiposity which prevents the uptake of glucose, inhibits lipolysis, and impairs lipogenesis. In hepatocytes, leptin induces insulin-like effects by regulating the insulin signalling pathway. Furthermore, elevated levels of leptin are overexpressed at a gene level in adipose tissue and are associated with leptin resistance, which is implicated in obesity, inflammation, and breast tumourigenesis [3]. Therefore, the increased leptin levels observed in the high fat diet group, further confirm the state of obesity.

Furthermore, the DIO phenotype observed in mice fed a HFD was corroborated by a significantly higher hepatic tissue weights observed in the HFD + NT + V and HFD + NT + DXR groups compared to the SD + NT + V and SD + T + DXR groups, respectively (Fig. 4). Our results correlate with a study conducted by Jung et al., 2013 [30] where C57BL6 mice fed a HFD for nine-weeks resulting in an increase in BM and liver mass gain.

### HFD and DXR promote macrovesicular steatosis in hepatocytes, but not fibrosis

As previously mentioned, the first structural changes that occurs when the liver is damaged is hydropic changes, where fluid filled vacuoles accumulates within the hepatocytes. It has been shown that a HFD can induce lipid accumulation via a process known as hepatic steatosis [27, 31]. Microvesicular steatosis was observed in the SD groups since hepatocytes are filled with tiny lipid droplets and the nucleus is located centrally in the cell. Macrovesicular steatosis was observed in the HFD groups where large fat droplets occupy the cytoplasm of hepatocytes, pushing the nucleus to the periphery (Fig. 5).

From the non-alcoholic fatty liver disease activity score (Table 1), > 66% steatosis was present within hepatocytes in the HFD + T + DXR group accompanied with lobular inflammation (score 2) and prominent ballooning (score 2), sinusoidal dilation and congestion, fatty infiltration, and ballooning of hepatocytes compared to the SD + NT + V (control) group where areas of steatosis alternated with areas of normal morphology (Fig. 5). In the SD + T + DXR group, micro-vesicular steatosis was observed, with 5%-33% steatosis in the hepatocytes. In the HFD + NT + DXR group, > 33%-66% of the hepatocytes had steatosis compared to the HFD + NT + V group. This indicates that DXR promotes hepatic steatosis in obesity.

Our histopathology observations correlate with previous studies conducted where a HFD and DXR treatment induced macrovesicular steatosis, ballooned hepatocytes, inflammatory cell infiltration and sinusoidal dilation, respectively [12, 27, 29, 32]. No significant fibrosis was observed in our experimental groups (Fig. 8). This indicates that the liver did not progress to cirrhosis, indicative of end-stage liver disease and that the highly metabolic organ had the capacity to regenerate itself or reverse the harmful stimulus in the broad spectrum of liver disease. This could also be attributed to the concentration and frequency of the DXR treatment as observed by other studies [32].

### HFD promotes lipid accumulation within hepatocytes and induce hepatotoxicity in combination with DXR treatment

The liver plays an important role in lipid metabolism and stores fatty acids in the form of triglycerides. It has been shown that a HFD promotes lipid accumulation within hepatocytes via hepatic steatosis resulting in disturbances in lipid metabolism [27, 31]. Due to image evaluation of Oil Red O-stained fresh tissue sections, our results indicated that the relative number of red pixels are highly significantly increased in the HFD groups compared to mice fed a SD (Fig. 6). In the present study, the relative number of red pixels are highly significantly increased in the HFD + T + DXR compared to the SD + T + DXR group (Fig. 6). Our results correlate with previous studies conducted by Layman et al., 2019 [27] & Tsuru et al., 2020 [33] where a HFD induced hepatic lipid accumulation and inflammation within hepatocytes of mice fed a HFD, resulting in hepatic steatosis. It is evident from our H&E and Oil Red O results that lipid accumulation was elevated in the HFD groups and specifically severe in the HFD + T + DXR group, which indicates that obesity and the tumour present in combination with DXR treatment induced hepatic steatosis, which is a hallmark of NAFLD (Figs. 5 and 7). We therefore conclude that hepatic steatosis (> 66% steatosis) occurred in the obese tumour-bearing mouse model, resulting in NAFLD. Our findings correlate with a study done by Layman et al., 2019 [27] where mice fed a HFD induced hepatic steatosis, within hepatocytes.

## Conclusion

**Fig. 9:**
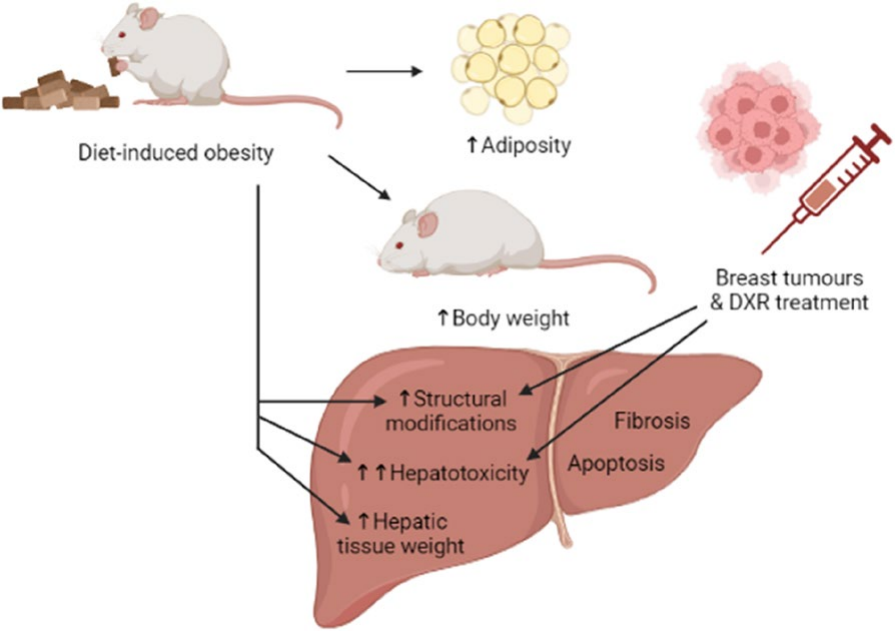
Summary of schematic representation of DXR-induced hepatotoxicity in an obese-tumour bearing mouse model. Demonstrating the effects of diet-induced obesity, breast tumours and DXR treatment on the outcomes of hepatotoxicity. Diet-induced obesity resulted in an increase in adiposity, body weight, and hepatic tissue weight in mice fed a HFD. Diet-induced obesity in the presence of breast tumours treated with DXR caused significant structural modifications to hepatic tissue and exacerbated the hepatoxicity in the obese tumour bearing mice. There was no evidence of fibrosis which indicates that the livers did not progress to cirrhosis

Our histopathology results indicated that hepatic tissues of mice fed a high fat diet and treated with DXR displayed severe histological damage, such as macrovesicular steatosis, sinusoidal dilation, and lobular inflammation. This indicates that more hepatic toxicity occurred in mice fed a high fat diet and treated with DXR. We therefore conclude that hepatic steatosis, a hallmark of NAFLD occurred in the obese tumour-bearing mouse model. There was no evidence of fibrosis which indicates that the livers did not progress to cirrhosis. This study showed that hepatotoxicity is aggravated in obesity as an underlying co-morbidity in breast cancer patients receiving adjuvant chemotherapy, such as DXR (Fig. 9). It has been shown that obesity is associated with poor clinical outcomes in patients receiving neo-adjuvant chemotherapy treatment regimens [34]. Lower doses of DXR are prescribed for obese cancer patients to reduce side effects and adverse toxicities, which could compromise drug efficacy and contribute to the development of resistance [34–36]. This study, therefore, provided clear evidence of the effects of DXR and the contributing role of obesity in the context of breast cancer.

## Supplementary Information

**Additional file 1: Fig. S1.** Original blots of total protein and caspase-9 (*n* = 1-4). **Fig. S2.** Original blots of total protein and caspase-8 (*n* = 1-4). **Fig. S3.** Original blots of total protein and cleaved caspase-8 (*n* = 1-4). **Fig. S4.** Original blots of total protein and caspase-3 (*n* = 1-4). **Fig. S5.** Original blots of total protein and cleaved PARP (*n* = 1-4). **Fig. S6.** Original blots of total protein and ALT (*n* = 1-4). **Table S1.** Dietary composition of the standard and high fat diets [16]. **Table S2.** Primary and secondary antibodies details.

## Data Availability

The datasets generated and analysed during the current study are not publicly available due datasets being too large to include but are available from the corresponding author on reasonable request.

## Abbreviations

ALT: Alanine transaminase
ANOVA: Analysis of variance
ARRIVE: Animal Research: Reporting of In Vivo Experiments
AST: Aspartate aminotransferase
BM: Body Mass
DNA: Deoxyribonucleic acid
DIO: Diet-induced obesity
DXR: Doxorubicin
ECL: Electrochemiluminescence
FFPE: Formalin fixed paraffin embedded
HBSS: Hank’s balanced salt solution
H&E: Haematoxylin & eosin
HFD: High fat diet
IVC: Individually ventilated cages
IL-6: Interleukin-6
JNK: Jun N-terminal kinase
MCP-1: Monocyte chemoattractant protein-1
NAFLD: Non-alcoholic fatty liver disease
NAS: Non-alcoholic fatty liver disease activity score
NF-kB: Nuclear factor kappa-light-chain-enhancer of activated B cells
NT: Non-Tumour
PVDF: Polyvinylidene difluoride
RES: Reticuloendothelial system
RIP1: Receptor-interacting protein 1
RIPA: Radio-immunoprecipitation assay buffer
ROS: Reactive oxygen species
RT: Room Temperature
SD: Standard diet
SDS: Sodium dodecyl sulfate
TBS-T: Tris-buffered saline tween
T2DM: Type 2 Diabetes Mellitus
T: Tumour
Top 2: Topoisomerase II
V: Vechile
