# Correction to: Obese mammary tumour‑bearing mice are highly sensitive to doxorubicin‑induced hepatotoxicity

**DOI:** 10.1186/s12885-026-15704-0

**Published:** 2026-02-13

**Authors:** Megan Sedeman, Claudia Christowitz, Louis de Jager, Anna‑Mart Engelbrecht

**Affiliations:** 1 Department of Physiological Sciences, Stellenbosch University, Stellenbosch Campus, Stellenbosch, South Africa; 2 Department of Global Health, Faculty of Medicine and Health Sciences, African Cancer Institute (ACI), Stellenbosch University, Cape Town, 8000 South Africa; 3 Division of Anatomical Pathology, Stellenbosch University and National Health Laboratory Service (NHLS), Tygerberg Hospital, Cape Town, 8000 South Africa; 4 Anatomical Pathology, PathCare, Cape Town, South Africa

**Correction to: BMC Cancer 22, 1240 (2022)**

**https://doi.org/10.1186/s12885-022-10189-z**

Following publication of the original article [1], an error was identified in **Fig. 7**. The microscopy image representing the **HFD + NT+DXR** group was inadvertently duplicated during figure preparation and does not correspond to the correct original image for this experimental group.

The error occurred during figure assembly, during which multiple microscopy images were generated and reviewed, and an incorrect panel was mistakenly included in the final published version. The figure has now been corrected by replacing the duplicated panel with the appropriate original microscopy image for the **HFD + NT+DXR** group. Original image dimensions have been retained to ensure accurate representation.

This correction does not affect the results, interpretation, or conclusions of the article. The original article has been corrected.

The authors apologise for this error.

Correct Fig. 7.


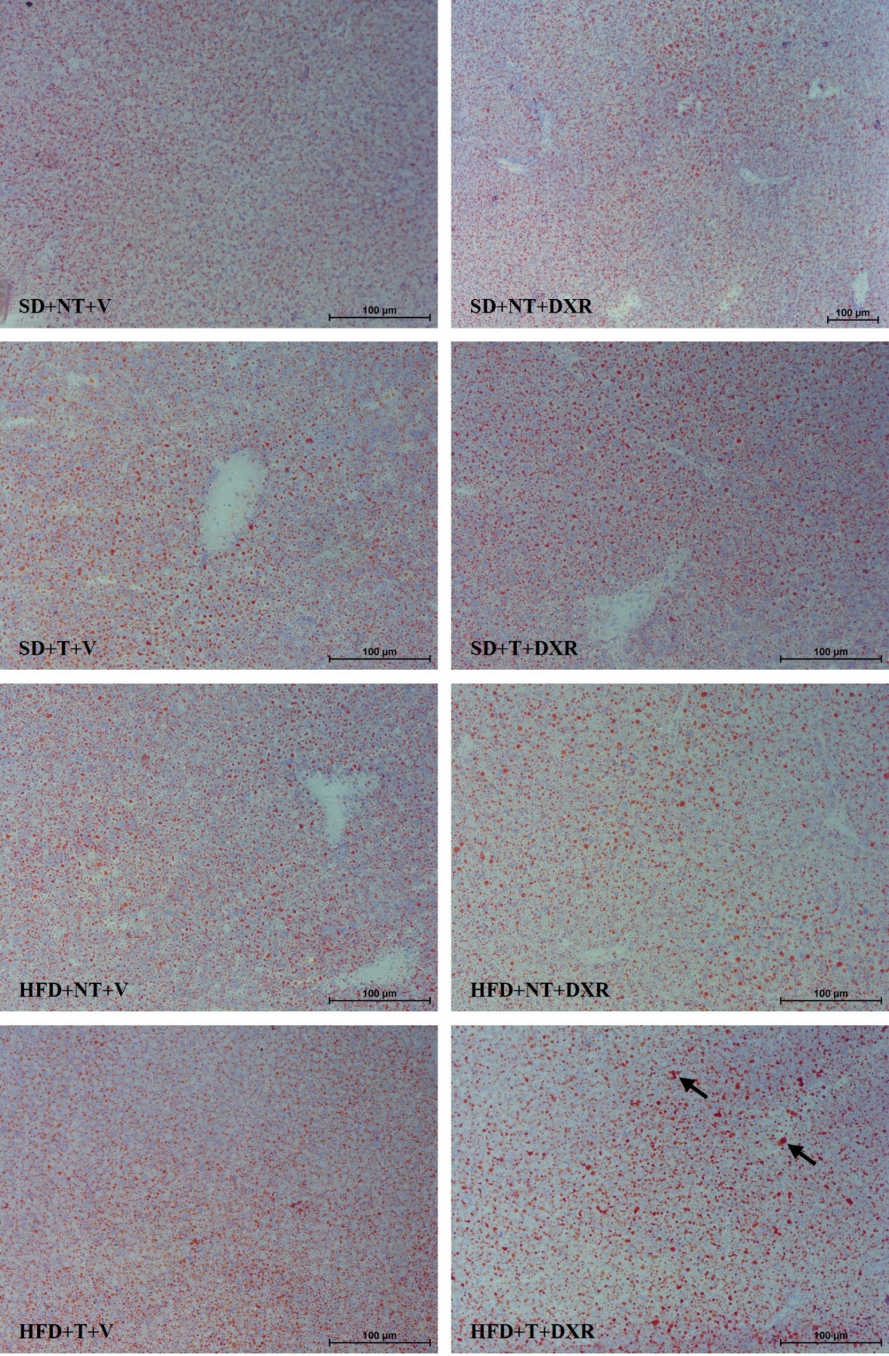


**Author approval statement**.

All authors have reviewed and approved the corrected Fig. 7 and this correction notice and agree to its publication.
