# Supplementary material for: Obese mammary tumour-bearing mice are highly sensitive to doxorubicin-induced hepatotoxicity
Source: BMC Cancer. 2022 Nov 30;22:1240. doi: 10.1186/s12885-022-10189-z (PMC9710042; doi:10.1186/s12885-022-10189-z)
Supplement: Supplementary file 1 — Additional file 1: Fig. S1. Original blots of total protein and caspase-9 (n = 1-4). Fig. S2. Original blots of total protein and caspase-8 (n = 1-4). Fig. S3. Original blots of total protein and cleaved caspase-8 (n = 1-4). Fig. S4. Original blots of total protein and caspase-3 (n = 1-4). Fig. S5. Original blots of total protein and cleaved PARP (n = 1-4). Fig. S6. Original blots of total protein and ALT (n = 1-4). Table S1. Dietary composition of the standard and high fat diets [16]. Table S2. Primary and secondary antibodies details. [file 12885_2022_10189_MOESM1_ESM.docx]

**Additional file: Supplementary data**

**Original blots of western blot analysis**

Below images indicate original full-length blots of all samples. Overexposure of non-specific bands were sometimes required to obtain optimal intensities for the bands of interest (based on the size (kDa) of the protein).

**
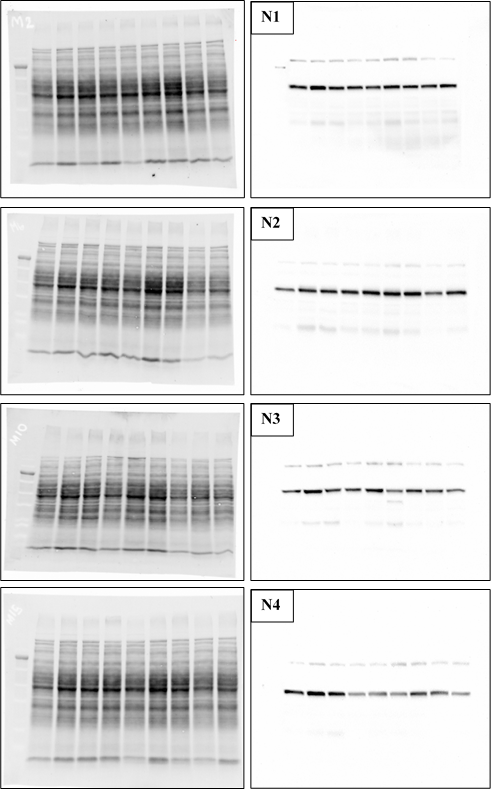
**

**Fig. S1:** **Original blots of total protein and caspase-9 (n = 1-4).** Lane 1: Standard sample (used for normalization); Lane 2: SD+NT+V; Lane 3: SD+NT+DXR; Lane 4: SD+T+V; Lane 5: SD+T+DXR; Lane 6: HFD+NT+V; Lane 7: HFD+NT+DXR; Lane 8: HFD+T+V; Lane 9: HFD+T+DXR.

**
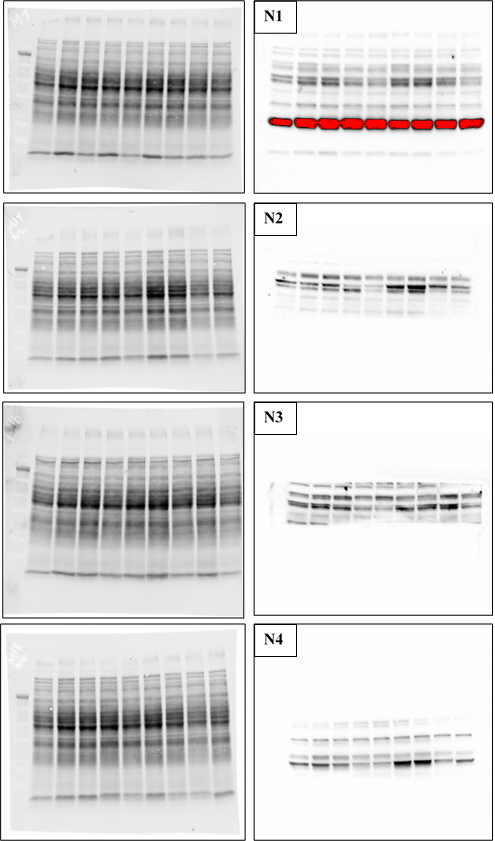
**

**Fig. S2: Original blots of total protein and caspase-8 (n = 1-4).** Lane 1: Standard sample (used for normalization); Lane 2: SD+NT+V; Lane 3: SD+NT+DXR; Lane 4: SD+T+V; Lane 5: SD+T+DXR; Lane 6: HFD+NT+V; Lane 7: HFD+NT+DXR; Lane 8: HFD+T+V; Lane 9: HFD+T+DXR.

**
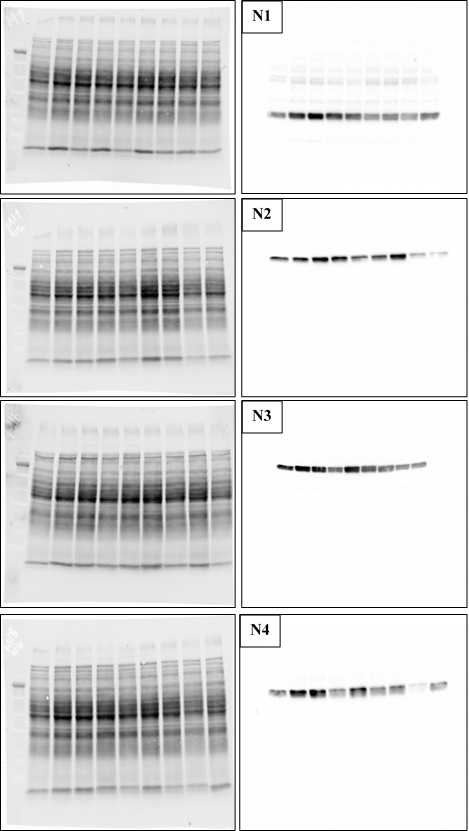
**

**Fig. S3: Original blots of total protein and cleaved caspase-8 (n = 1-4).** Lane 1: Standard sample (used for normalization); Lane 2: SD+NT+V; Lane 3: SD+NT+DXR; Lane 4: SD+T+V; Lane 5: SD+T+DXR; Lane 6: HFD+NT+V; Lane 7: HFD+NT+DXR; Lane 8: HFD+T+V; Lane 9: HFD+T+DXR.

**
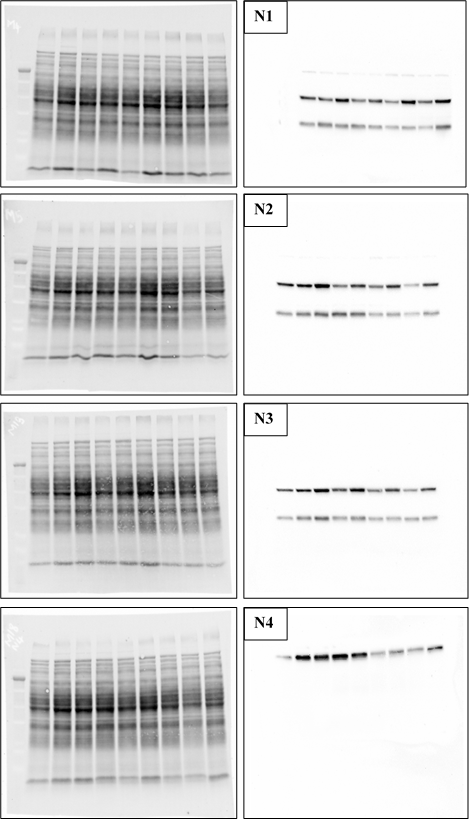
**

**Fig. S4: Original blots of total protein and caspase-3 (n = 1-4).** Lane 1: Standard sample (used for normalization); Lane 2: SD+NT+V; Lane 3: SD+NT+DXR; Lane 4: SD+T+V; Lane 5: SD+T+DXR; Lane 6: HFD+NT+V; Lane 7: HFD+NT+DXR; Lane 8: HFD+T+V; Lane 9: HFD+T+DXR.

**
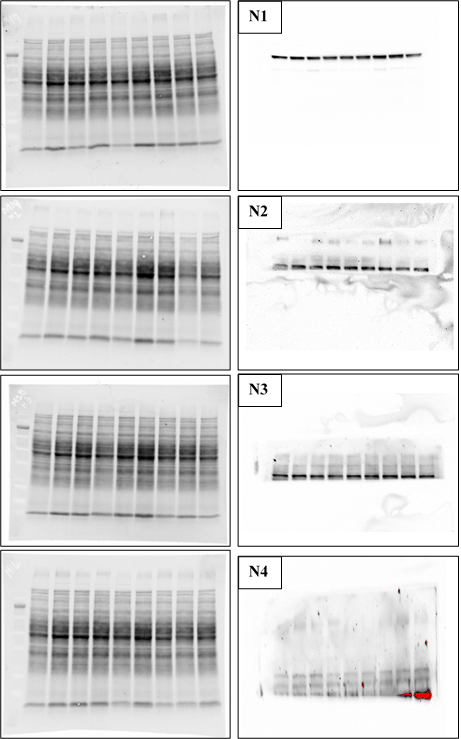
**

**Fig. S5: Original blots of total protein and cleaved PARP (n = 1-4).** Lane 1: Standard sample (used for normalization); Lane 2: SD+NT+V; Lane 3: SD+NT+DXR; Lane 4: SD+T+V; Lane 5: SD+T+DXR; Lane 6: HFD+NT+V; Lane 7: HFD+NT+DXR; Lane 8: HFD+T+V; Lane 9: HFD+T+DXR

**
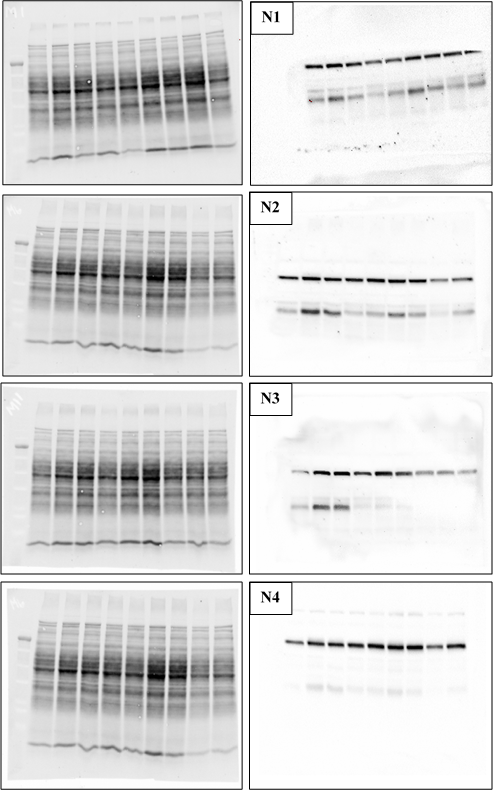
**

**Fig. S6: Original blots of total protein and ALT (n = 1-4).** Lane 1: Standard sample (used for normalization); Lane 2: SD+NT+V; Lane 3: SD+NT+DXR; Lane 4: SD+T+V; Lane 5: SD+T+DXR; Lane 6: HFD+NT+V; Lane 7: HFD+NT+DXR; Lane 8: HFD+T+V; Lane 9: HFD+T+DXR

**Table S1. Dietary composition of the standard and high fat diets** [16].

|  | **Standard diet (D12450J)** | | **High fat diet (D12492)** | |
| --- | --- | --- | --- | --- |
| **Diet component** | **gram %** | **kcal %** | **gram %** | **kcal %** |
| Protein | 19.2 | 20 | 26.2 | 20 |
| Carbohydrates | 67.3 | 70 | 26.3 | 20 |
| Fat | 4.3 | 10 | 34.9 | 60 |
| Total |  | 100 |  | 100 |
| Kcal/gram | 3.85 |  | 5.24 |  |
| **Ingredients** | **gram** | **kcal** | **gram** | **kcal** |
| Casein, 30 Mesh 200 | 200 | 800 | 200 | 800 |
| L-Cystine | 3 | 12 | 3 | 12 |
| Corn Starch | 506.2 | 2024.8 | 0 | 0 |
| Maltodextrin 10 | 125 | 500 | 125 | 500 |
| Sucrose | 68.8 | 275.2 | 68.8 | 275.2 |
| Cellulose BW200 | 50 | 0 | 50 | 0 |
| Soybean Oil | 25 | 225 | 25 | 225 |
| Lard* | 20 | 180 | 245 | 2205 |
| Mineral Mix S10026 | 10 | 0 | 10 | 0 |
| Dicalcium Phosphate | 13 | 0 | 13 | 0 |
| Calcium Carbonate | 5.5 | 0 | 5.5 | 0 |
| Potassium Citrate, 1 H_2_O | 6.5 | 0 | 6.5 | 0 |
| Vitamin Mix V10001 | 10 | 40 | 10 | 40 |
| Choline Bitartrate | 2 | 0 | 2 | 0 |
| FD&C Yellow Dye #5 | 0.04 | 0 |  |  |
| FD&C Blue Dye #1 | 0.01 | 0 | 0.05 | 0 |
| **Total** | **1055.05** | **4057** | **773.85** | **4057** |
| Cholesterol (mg)/4057 kcal | - | 54.4 | - | 216.4 |
| Cholesterol (mg)/kg | - | 51.6 | - | 279.6 |

*As per manufacturer product data sheet (Research diet Inc., New Jersey, USA).*

**Table S2. Primary and secondary antibodies details.** Abbreviations: CST (Cell Signaling Technology); PARP (Poly (ADP-ribose) polymerase); ALT (Alanine Transaminase); HRP (horseradish peroxidase); SCBT (Santa Cruz Biotechnology).

| **Primary antibody**  **(1° AB)** | **Company (Catalogue number 1°AB)** | **Specie** | **1° AB Dilution** | **Molecular weight** | **Secondary Antibody (2° AB)** | **Company (Catalogue number 2°AB)** | **2° AB Dilution** |
| --- | --- | --- | --- | --- | --- | --- | --- |
| Caspase-8 | Abcam (ab25901) | Rabbit | 1: 1000 | 18, 57 kDa | Anti-Rabbit IgG HRP-linked | CST  (7074S) | 1:10 000 |
| Caspase-9 | CST  (9508) | Mouse | 1: 1000 | 47, 35 kDa | Anti-Mouse IgG HRP-linked | CST  (7076S) | 1:10 000 |
| Caspase-3 | Abcam (ab184787) | Rabbit | 1: 1000 | 17, 32 kDa | Anti-Rabbit IgG HRP-linked | CST  (7074S) | 1:10 000 |
| PARP-1 | Abcam (ab191217) | Rabbit | 1: 1000 | 89, 116 kDa | Anti-Rabbit IgG HRP-linked | CST  (7074S) | 1:10 000 |
| ALT | SCBT  (Sc-271861) | Mouse | 1: 1000 | 48 kDa | Anti-Mouse IgG HRP-linked | CST  (7076S) | 1:10 000 |
